# A systematic review and meta‐analysis of mobile health application interventions for community‐dwelling older adults with mild cognitive impairment and dementia

**DOI:** 10.1002/alz.085504

**Published:** 2025-01-09

**Authors:** Natalie Jian‐Ting Wee, Penny Lun, Liang Guo, Luming Shi, Edwin Shih‐Yen Chan, Yiew Hock James Low

**Affiliations:** ^1^ Khoo Teck Puat Hospital, Singapore Singapore; ^2^ Geriatric Education and Research Institute, Singapore Singapore; ^3^ Singapore Clinical Research Institute, Consortium for Clinical Research and Innovation, Singapore Singapore; ^4^ Cochrane Singapore, Singapore Singapore; ^5^ Duke‐NUS Medical School, Singapore Singapore

## Abstract

**Background:**

Mobile health applications have the potential to enhance dementia care and promote well‐being among older adults living independently. This systematic review aims to synthesise and evaluate the existing evidence on the effectiveness of mobile applications developed to improve or maintain cognitive function among older adults diagnosed with mild cognitive impairment (MCI) and dementia.

**Method:**

A systematic search was conducted across major electronic databases, including PubMed, The Cochrane Library, Embase, and PsycInfo, to identify relevant studies published from 2012 to 2023. Inclusion criteria encompassed randomised controlled trials and observational studies that assessed the impact of mobile applications on cognitive function in individuals with MCI or dementia compared with standard clinical care. Meta‐analysis was conducted to quantify the effect of cognitive training using mobile applications on cognitive outcomes, with subgroup analysis to explore the potential difference between various types of interventions.

**Result:**

A total of 22 studies with 1227 patients (mean age range from 65 to 84) were included out of 5774 articles initially identified from the search. The included studies reported mobile applications with diverse cognitive training interventions applications run on a variety of digital platforms, such as mobile phones, tablets and laptops. A meta‐analysis was conducted to quantify the overall effect of cognitive training using mobile applications on cognitive function, employing standardized mean differences and random‐effects models.

Preliminary findings suggest a positive effect of mobile health applications in improving cognitive function in cognitively‐impaired older adults. Subgroup analysis explored the impact of different types of cognitive interventions on the observed effects.

**Conclusion:**

Mobile applications improve cognitive function compared to standard clinical care in community‐dwelling older adults with cognitive impairment. This systematic review contributes to the understanding of the efficacy of cognitive training delivered via mobile applications for individuals with dementia. The synthesis of available evidence provides insights into the potential benefits of specific interventions and highlights gaps in the current literature. The results of this review have implications for the development of mobile applications to target cognitive function, and the design of future research studies aimed at assessing the use of mobile applications in individuals with dementia.

